# Choline Dehydrogenase Polymorphism rs12676 Is a Functional Variation and Is Associated with Changes in Human Sperm Cell Function

**DOI:** 10.1371/journal.pone.0036047

**Published:** 2012-04-27

**Authors:** Amy R. Johnson, Sai Lao, Tongwen Wang, Joseph A. Galanko, Steven H. Zeisel

**Affiliations:** 1 Department of Nutrition, University of North Carolina at Chapel Hill, Chapel Hill, North Carolina, United States of America; 2 Department of Medicine, School of Medicine, University of North Carolina at Chapel Hill, Chapel Hill, North Carolina, United States of America; 3 Nutrition Research Institute, Gillings School of Global Public Health and School of Medicine, University of North Carolina at Chapel Hill, Chapel Hill, North Carolina, United States of America; University of Otago, New Zealand

## Abstract

Approximately 15% of couples are affected by infertility and up to half of these cases arise from male factor infertility. Unidentified genetic aberrations such as chromosomal deletions, translocations and single nucleotide polymorphisms (SNPs) may be the underlying cause of many cases of idiopathic male infertility. Deletion of the choline dehydrogenase (*Chdh*) gene in mice results in decreased male fertility due to diminished sperm motility; sperm from *Chdh^−/−^* males have decreased ATP concentrations likely stemming from abnormal sperm mitochondrial morphology and function in these cells. Several SNPs have been identified in the human *CHDH* gene that may result in altered CHDH enzymatic activity. rs12676 (G233T), a non-synonymous SNP located in the *CHDH* coding region, is associated with increased susceptibility to dietary choline deficiency and risk of breast cancer. We now report evidence that this SNP is also associated with altered sperm motility patterns and dysmorphic mitochondrial structure in sperm. Sperm produced by men who are GT or TT for rs12676 have 40% and 73% lower ATP concentrations, respectively, in their sperm. rs12676 is associated with decreased CHDH protein in sperm and hepatocytes. A second SNP located in the coding region of *IL17BR*, rs1025689, is linked to altered sperm motility characteristics and changes in choline metabolite concentrations in sperm.

## Introduction

An estimated 15% of couples are affected by infertility [1] and male factor infertility is the suspected cause in 30–50% of these couples. Although the exact cause of many of these cases are unknown, chromosomal deletions, translocations and SNPs are associated with infertility in as many as 30% of these men [2]. Identifying and understanding how genetic anomalies affect spermatogenesis and fertilization will improve the likelihood of overcoming male infertility. Conversely, effective male contraception may be developed based on data showing associations between genetic variation, nutrient metabolism and male factor infertility.

Although the relationship between overall nutritional status and reproduction is well documented [3], [4], [5], the relationship between micronutrient metabolism and reproduction is understudied. There is some evidence that aberrant micronutrient metabolism may play a causative role in male factor infertility. Dietary deficiencies of vitamins A, C and E as well as trace metals such as zinc and selenium are associated with male infertility in animals and humans [6], [7], [8], [9], [10], [11], [12], [13], [14], [15], [16], [17] Choline is an essential nutrient for humans [18] and is important for normal fetal development [19]. A link between choline metabolism and male fertility has been demonstrated in only one paper [20]. Geer reported both normal mating behavior and sperm motility required adequate choline availability in Drosophila melanogaster and that carnitine, a proposed choline substitute, was unable to support male fertility. We discovered that choline dehydrogenase (CHDH, EC 1.1.99.1) is necessary for normal male fertility in mice [21]. Male choline dehydrogenase knockout mice (*Chdh^−/−^*) are infertile due to severely compromised sperm motility; decreased motility is the result of abnormal mitochondrial structure and function in sperm cells.

Betaine (*N,N,N*-trimethylglycine), a metabolite of choline, donates methyl groups for the formation of methionine from homocysteine and is an organic osmolyte used by cells for regulatory volume control [22]. Dietary sources of betaine include wheat, shellfish, spinach and sugar beets [23], [24]. In addition, betaine can be made *de novo* via the oxidation of choline in a series of reactions catalyzed by CHDH and betaine aldehyde dehydrogenase (BADH, EC 1.2.1.8) [25], [26], [27], [28], [29], [30]. Conversion of choline to betaine takes place in the mitochondrial matrix following the transport of choline across mitochondrial membranes [31], [32], [33]. The betaine formed is a zwitterion at neutral pH and diffuses out of mitochondria for use in one-carbon metabolism [34].

SNPs have been identified in the human *CHDH* gene; rs12676 (G233T) is a non-synonymous SNP located in exon 3 of the gene. Occurrence of the variant T allele results in the replacement of arginine, a polar, hydrophilic amino acid, with leucine, a hydrophobic amino acid. 38–40% of individuals are heterozygous and 2–9% are homozygous for rs12676 [35], [36]. The *CHDH* minor T allele is associated with increased susceptibility to developing clinical symptoms of dietary choline deficiency (steatosis and muscle cell damage) [36] as well as increased risk of breast cancer [35]. Although not in the *CHDH* gene, a SNP in the adjacent interleukin 17 beta receptor (*IL17βR*) gene, rs1025689, is associated with increased risk of developing choline deficiency specifically in men (odds ratio 13.5; see [36] for detailed description of this study). We now present data indicating that rs12676 genotype is also associated with dysmorphic mitochondrial structure, changes in sperm motility patterns and decreased energy status in human sperm. Sperm and hepatocytes harboring the TT rs12676 genotype have less CHDH protein, suggesting that this SNP is functional. In addition, rs1025689 is linked to changes in sperm motility patterns, suggesting that these SNPs may be contributing factors in the occurrence of idiopathic male infertility.

## Materials and Methods

### Chemicals and Reagents

All chemicals and reagents used were obtained from Sigma Aldrich (St. Louis, MO), unless otherwise noted.

### Ethics statement

The study design and all procedures used in this study were approved by the University of North Carolina at Chapel Hill Office of Human Research Ethics Institutional Review Board. Subjects were at least 18 years of age and were recruited from the Charlotte-Kannapolis, North Carolina region. Informed written consent was obtained from all subjects at the initial clinic visit. Subjects were compensated up to $125 for their participation in the study.

### Study design and recruitment

This study was conducted at the University of North Carolina at Chapel Hill Nutrition Research Institute (Kannapolis, North Carolina) and was implemented in two phases – screening subjects for rs12676 and rs1025689 genotype, and analysis of semen and sperm specimens collected from subjects with these SNPs. Subjects were screened to determine the frequency of these SNPS in our study population and to allow for enrichment of the homozygous variant genotypes. Participants were recruited via mass email, advertisements in Craig's list, newspaper articles describing the study and in-person recruiting at local community colleges.

### Blood collection and DNA isolation

Subjects were asked to complete the health questionnaire form (Figure S1) at the screening visit. In order to screen subjects for the rs12676 and rs1025689 SNPs, blood was collected into Vacutainer Cell Preparation Tubes (CPT tubes; BD Diagnostics, Franklin Lakes, NJ) containing sodium citrate; lymphocytes were separated from other blood components for subsequent genomic DNA extraction. All CPT tubes were stored on ice if not processed immediately; all CPT tubes were processed within 1 hour of sample collection. Briefly, CPT tubes were centrifuged in a Sorvall RC-3B centrifuge equipped with an H-2000B rotor (Thermo Fisher Scientific, Waltham, MA) at 1500× *g* for 30 minutes at room temperature. Plasma was aliquoted into 2 mL microfuge tubes and stored at −80°C for choline metabolite analysis. The lymphocyte layer was washed with phosphate buffered saline (PBS), transferred to a 15 mL conical tube and was pelleted by centrifugation at 1000× *g* for 5 minutes at room temperature. Pellets were again washed with PBS, transferred to 1.5 mL microfuge tubes and pelleted by centrifugation in an Eppendorf 5415D microcentrifuge at 800× *g* for 5 minutes at room temperature.

Genomic DNA was purified from lymphocyte pellets using a QIAamp DNA Mini Kit (Qiagen, Valencia, CA) according to manufacturer's instructions with some modification. Specifically, lymphocyte pellets were equilibrated to room temperature and resuspended in 500 µL PBS. The amounts of Qiagen Protease, Buffer AL and ethanol (96%–100%) were adjusted proportionally as indicated by the manufacturer's instructions. Two separate elutions of 100 µL with Qiagen Buffer AE were performed. Samples were incubated at room temperature for 5 minutes prior to each elution. Both elutions were collected in the same 1.5 mL microfuge tube for a final volume of 200 µL. DNA quality and concentration was determined using a Nanodrop 8000 Spectrophotometer (Thermo Scientific, Wilmington, DE).

### rs12676 genotyping

rs12676 genotype was determined by direct sequencing. A 260 base pair region of the *CHDH* gene containing rs12676 was PCR amplified using Deep Vent_R_™ (exo-) DNA polymerase (New England Biolabs, Ipswich, MA) according to manufacturer's recommendations. The primers used for amplification were: *CHDH forward* 5′-ATTCCCCTCCGTGGATCAG-3′ and *CHDH reverse* 5′-TGTCGTCGCACAGGTTGG-3′. Each 50 µL reaction contained 600 ng of genomic DNA, primers at a final concentration of 200 nM, and 4 units of Deep Vent_R_™ (exo-) DNA polymerase. The PCR conditions were: an initial denaturing step at 98°C for 10 minutes followed by 30 cycles of denaturing at 98°C for 1 minute and annealing/extension at 72°C for 5 minutes. PCR products were purified from other reaction components using a QIAquick PCR Purification Kit (Qiagen, Valencia, CA) and the resulting DNA concentration was determined using Nanodrop 8000 Spectrophotometer (Thermo Scientific, Wilmington, DE). Purified *CHDH* fragments were sequenced using BigDye® Terminator chemistries (Applied Biosystems, Carlsbad, CA) by Eton Bioscience, Inc (Research Triangle Park, NC) using the *CHDH forward* primer. rs12676 genotype was determined by examining sequencing chromatograms using Sequence Scanner software (version 1.0, Applied Biosystems, Carlsbad, CA).

### rs1025689 genotyping

rs1025689 genotype was determined using a TaqMan® SNP genotyping assay (Applied Biosystems, Carlsbad, CA) according to manufacturer's instructions. PCR reactions were performed using a StepOne™ Real Time PCR System and 2× TaqMan® Genotyping Master Mix (Applied Biosystems, Carlsbad, CA).

### Semen collection and processing

Subjects were asked to refrain from sexual activity for 48 hours prior to semen donation. Semen was produced by masturbation and collected into 50 mL sterile sample cups. Olive oil was provided as a lubricant to use as necessary. Samples were incubated at room temperature for 30 minutes to allow for liquefaction. Semen volume was measured with a pipette. Sperm were separated from other seminal fluid components by layering the sample over a 45% ISolate®/human tubular fluid (HTF; 190 mM NaCl, 9 mM KCl, 0.7 mM KH_2_PO_4_, 0.3 mM MgSO_4_-7H_2_0, 4 mM CaCl_2_ – 2H_2_0, 0.025 mM NaHCO_3_, 2.78 mM D-glucose, 21.4 mM lactate, 0.33 mM pyruvate and 5 mg/mL bovine serum albumin (BSA; Fraction V), 5 M NaCl was added as necessary to adjust osmolality) media gradient followed by centrifugation at 300× *g* for 20 minutes at room temperature using a Beckman-Coulter Allegra X-15R Centrifuge and SX4750A rotor. ISolate® was purchased from Irvine Scientific (Santa Ana, CA). The supernatant was discarded and the resulting sperm pellet was washed twice in 3 mL HTF followed by centrifugation at 300× *g* for 10 minutes at room temperature. Sperm were resuspended in 4 mL HTF and used for subsequent analyses.

### Sperm counts

The total number of sperm per ejaculate and sperm concentration were determined by counting cells with a hemocytometer.

### Sperm motility

Sperm were diluted 1∶10–1∶15 in HTF for motility measurements. 200 µL of diluted sperm were placed into a 35 mm glass bottom dish and covered with a coverslip. For each sample, video of 10 random, unique microscope frames were recorded using a Zeiss Axio Observer (Carl Zeiss, Inc, Thornwood, NY) inverted microscope equipped with a temperature controlled incubation chamber equilibrated to 37°C. Sperm were viewed under phase contrast conditions with a 20× objective lens.

Motility parameters including mean velocity (MVUS), curvilinear velocity (VCL), straight distance velocity (VSL) and mean tortuosity (MT; MT = VCL/VSL) were determined using Zeiss AxioVision (release 4.7) image tracking software (Carl Zeiss, Inc, Thornwood, NY) as previously described [37].

### Electron microscopy

Approximately 500 µL of washed sperm were transferred to 1.5 mL microfuge tubes; sperm were pelleted by centrifugation at 16,000× *g* for 5 minutes at room temperature. The supernatant was discarded and sperm pellets were fixed for transmission electron microscopy in 2% paraformaldehyde, 2.5% gluteraldehyde, 0.2% picric acid in 0.1 M sodium cacodylate, pH 7.2. The pellet was encapsulated in 2% agarose buffered with 0.1 M sodium cacodylate buffer, pH 7.2. The encapsulated pellet was post-fixed in 1% osmium tetroxide in 0.1 M sodium cacodylate buffer for 1 hour. Samples were washed in deionized water, dehydrated through an ethanol series, transferred to propylene oxide, infiltrated and embedded in Polybed 812 resin (Polysciences, Inc., Warrington, PA). 70 nm ultrathin sections were post-stained in 4% aqueous uranyl acetate and 0.4% lead citrate. Samples were examined and photographed using a Zeiss EM-10A transmission electron microscope (LEO Electron Microscopy, Thornwood, NY) with an accelerating voltage of 60 kV.

### ATP assay

ATP concentration in sperm was measured using an ATP Bioluminescence Assay Kit CLS II (Roche Diagnostics, Mannheim, Germany) according to manufacturer's instructions. Luminescence was measured using a 1420 VICTOR^2^ microplate reader (Perkin Elmer, Waltham, MA). ATP concentration was normalized to number of sperm analyzed.

### CHDH expression

Sperm pellets were lysed in a buffer of 2% w/v SDS, 0.375 M Tris, pH 6.8, 10% sucrose [38]. Lysates were boiled at 100°C for 5 minutes and clarified by centrifugation at 16.1× *g* for 10 minutes at 4°C. Protein concentration was determined by the BCA protein assay (Pierce/Thermo Scientific, Rockford, IL). 10 µg of protein lysate were resolved by SDS-PAGE and transferred to PVDF membrane (Bio-Rad, Hercules, CA). Primary human hepatocyte lots, screened for rs12676 genotype as described above, were purchased from Zen-Bio, Inc. (Research Triangle Park, NC). Three lots for each rs12676 genotype were obtained and lysates were made in radioimmunoprecipitation assay (RIPA) buffer and protein concentration measured using the BCA assay. Equal volumes of lysate (80–200 µg protein) were resolved by SDS-PAGE and transferred to PVDF as described above. The membrane was incubated with anti-CHDH antibody (1∶1000 dilution in 5% BSA/PBS-T; ProteinTech Group, Inc., Chicago, IL) at 4°C overnight. A 1∶10,000 dilution of HRP-conjugated anti-rabbit secondary antibody (Millipore, Billerica, MA) was incubated with the membrane for 1 hour at room temperature and bands detected with enhanced chemiluminescence (SuperSignal® West Pico ECL, Thermo Scientific, Rockford, IL). The number of pixels in the CHDH band was quantitated using the lasso tool in Adobe Photoshop (Adobe Photoshop CS3 Extended v.10.0.1) and the number of pixels per microgram of total protein loaded was calculated. As an external control for protein integrity, the abundance of ∝-TUBULIN (sperm) or ß-ACTIN protein (hepatocytes) was determined using mouse monoclonal antibodies (anti-alpha-tubulin antibody: 1∶2000 dilution in 5% BSA/PBST (Sigma Aldrich, St.Louis, MO); anti-beta-actin antibody: 1∶10,000 dilution in 5% BSA/PBS-T; Abcam, Cambridge, MA) and an HRP-conjugated goat anti-mouse secondary antibody (1∶5000 dilution in 5% BSA/PBS-T; Abcam, Cambridge, MA)

### Plasma and sperm choline metabolite analyses

The concentrations of choline and its metabolites [choline, betaine, glycerophosphocholine (GPCho), phosphatidylcholine (PtdCho), and sphingomyelin (SM)] in plasma and sperm were measured by liquid-chromatography ionization-isotope dilution mass spectrometry (LC-ESI-IDMS) as previously described [39]. Phosphocholine was not detected in either plasma or sperm. GPCho was not detected in plasma. SM was not detected in sperm. Plasma samples were collected from the entire screened population on Day 1. Three million sperm per subject were pelleted and flash frozen in liquid nitrogen on Day 2.

### Statistical analyses

Statistical differences among genotypes were determined using JMP 9.0 software (SAS Institute, Cary, NC) using ANOVA and Tukey-Kramer HSD. Statistical tests were performed on log 10 transformed data for semen volume, total sperm per ejaculate and sperm concentration as these data failed a Shapiro -Wilk test of normality. Only data from sperm recorded for at least 3 seconds were included in motility statistical analyses. In order to address the intra-individual variation in the sperm motility data the following method was used to determine statistical differences. For each continuous measure (MT, MVUS, VCL and VSL) cutpoints for quartiles were determined from all observations having the wild type/wild type (WW) genotype for that SNP. Then using those cutpoints, all observations were placed into a quartile and an association between SNP level and the most extreme quartiles (1 and 4) was assessed via a repeated measures logistic regression with quartile (1 or 4) as the response and SNP as the predictor. The repeated measures on subject were taken into account by using a compound symmetric correlation matrix for observations within the same subject. P-values less than 0.05 were considered statistically significant.

## Results

### rs12676 and rs1025689 distribution frequencies

rs12676 and rs1025689 distribution frequencies were calculated for the population of men screened for inclusion in this study. For rs12676, 52% of subjects were GG, 41% were GT and 7% were TT (Table 1). For rs102689, 22% were GG, 48% were GC and 30% were CC. These results are in agreement with published data regarding these two SNPs [35], [36], [40]. It is important to note that 100% of men who were homozygous for rs12676 had at least one minor C allele of rs1025689; 83% of men with the TT rs12676 genotype were also CC for rs1025689. In addition, 91% of men who were GT for rs12676 had at least 1 C allele for rs1025689.

**Table 1:** rs12676 and rs1025689 SNP distribution frequencies in the screened population.

rs12676 *(G→T)*	
GG	41 (52%)
GT	32 (41%)
TT	6 (7%)
rs1025689 *(G→C)*	
GG	17 (22%)
GC	38 (48%)
CC	24 (30%)

Allele distribution frequency was calculated as a percentage of the total screened population following genotyping by direct sequencing (rs12676) or TaqMan assay (rs1025689). N = 79.

### Study population

**Figure 1:**
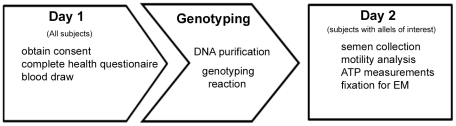
Study design. On Day 1, all subjects gave informed consent, completed a health questionnaire form and blood samples were collected. Plasma was separated from other blood components and reserved for measures of choline metabolites. Genomic DNA was isolated from purified lymphocytes and used to genotype subjects for rs12676 and rs1025689 SNPs. Individuals with the genotypes of interest were invited to complete Day 2 of the study which entailed leaving a semen sample for measures of semen characteristics, sperm motility, ATP concentration and mitochondrial morphology.

Average age, average number of biological children per subject and occurrence of abnormal semen characteristic or infertility were calculated from self-reported information provided by the health questionnaire form completed on Day 1 (Figure 1 and Figure S1). The average age of the entire screened population was 33.5 years and subject age ranged from 18 to 76 years. The average number of biological children per subject was calculated by dividing the total number of children per genotype by the number of subjects who answered this question (Table 2). Men who were TT for rs12676 reported the lowest number of biological children per subject (0.33) while men who were wild type for rs1025689 reported the highest (0.94). 33% (2 of 6) of men with rs12676 TT genotype reported semen abnormalities or infertility; these were reported by 9.7% (4 of 41) of men who were wild type for rs12676. For rs1025689, 11.7% (2 of 17) of GG, 5.2% (2 of 38) of GC and 8.3% (2 of 24) of CC subjects reported semen abnormalities or infertility.

**Table 2:** Characteristics of the study population.

	rs12676			rs1025689		
	G→T			G→C		
	GG	GT	TT	GG	GC	CC
	*(N = 41)*	*(N = 41)*	*(N = 41)*	*(N = 41)*	*(N = 41)*	*(N = 41)*
Average age (years)	33	32	36	39	33	28
*Age Range*	*18–76*	*18–56*	*18–58*	*19–76*	*18–58*	*18–58*
Average number biological children/subject	0.80	0.78	0.40	0.94	0.70	0.61
Cases of semen abnormalities/infertility	4	0	2	2	2	2
*Percent of group reporting semen abnormality/infertility*	*9.7%*	*0%*	*33%*	*11.7%*	*5.2%*	*8.3%*

Average age, average number of biological children per subject and occurrence of abnormal semen characteristics or infertility were calculated from self-reported information. N = 79, except for average number of biological children, which was calculated only in men who answered this question (N = 71).

### Semen parameters

rs12676 genotype was not associated with changes in semen volume, number of sperm per ejaculate or sperm concentration (Table 3). Men who were homozygous for rs1025689 had decreased sperm concentration compared to men who were heterozygous for this SNP. Mean values for these parameters were all within the normal range expected of the general human population [41]. All results were above the World Health Organization 5% lower reference limit for semen parameters from the general population except for total sperm per ejaculate and sperm concentration for men who were CC for rs1025689 [41].

**Table 3:** Semen parameters by rs12676 and rs1025689 genotype.

		rs12676			rs1025689		
Parameter	Normal Range†	GG	GT	TT	GG	GC	CC
	*(5% WHO lower limit)* †						
**Semen volume (mL)**	0.8–7.0	1.9±0.3	2.5±0.4	2.4±0.8	1.6±2.2	2.8±0.4	1.9±0.3
	*(1.2)*						
**Total sperm (10^6^)**	11–772	21.9±6.9	48.9±16.7	30.6±24.5	24.9±8.8	50.7±15.9	16.4±11.3
	*(20)*						
**Sperm concentration (10^6^/mL)**	4–237	9.7±2.6	22.0±6.6	11.2±8.0	14.4±4.0	20.9±6.3	6.7±3.7*
	*(9)*						

Semen volume, total number of sperm per ejaculate and sperm concentration was determined by measuring the volume of the ejaculate before washing and counting the number of sperm using a hemocytometer. For rs12676 *N* = 19 (GG), 22 (GT) or 5 (TT). For rs1025689 *N* = 12 (GG), 22 (GT), 11 (CC). Values are mean ± SEM. Statistical differences among groups were tested on log 10 transformed data using ANOVA and Tukey-Kramer HSD.

* indicates P<0.05.

† World Health Organization reference values and 5% lower reference limit for human semen characteristics within the general population.

### Sperm motility characteristics

**Figure 2:**
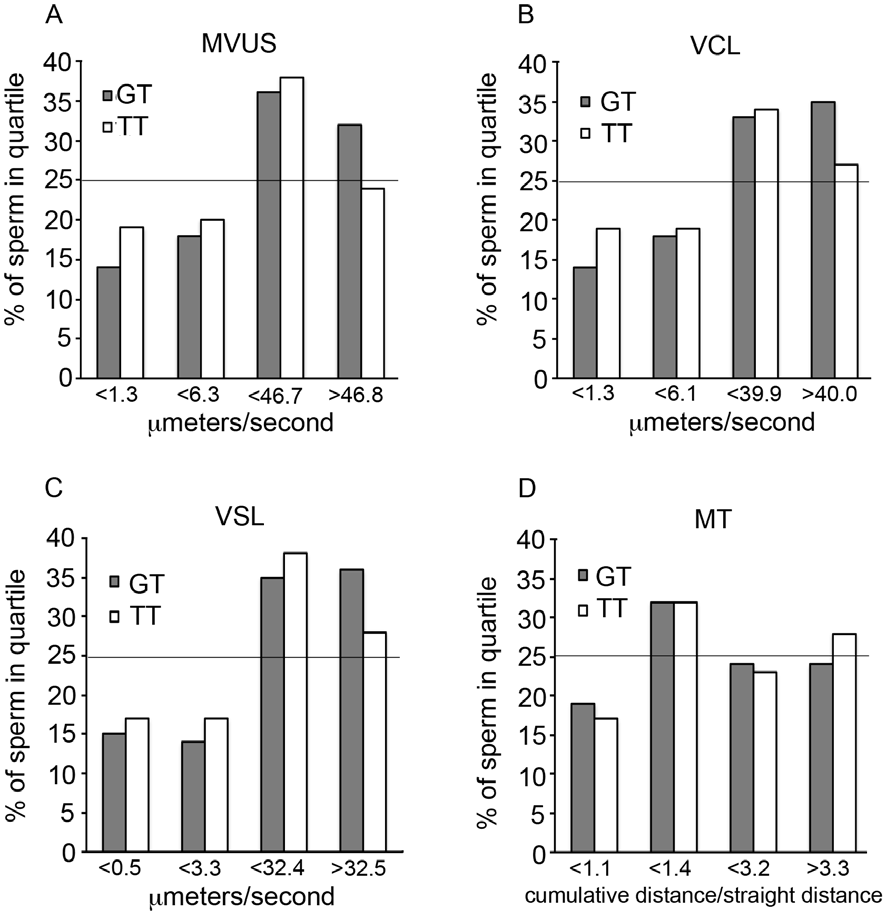
Motility characteristics of sperm change with rs12676 genotype. Mean velocity (MVUS, *A*), curvilinear velocity (VCL, *B*), straight line velocity (VSL, *C*) and mean tortuosity (MT, *D*) were determined for sperm. Sperm were prepared and motility characteristics measured as described in the Methods section. N = 2756 (GG), 2553 (GT), 827 (TT) sperm. Statistical analyses were performed as described in the Methods section; refer to Table 4 for p-values. X-axis values indicate the upper limit of each quartile; the line at 25% represents values for sperm from GG subjects.

Sperm from men who were homozygous for rs12676 had increased curvilinear velocity and tortuosity when compared to sperm from men who were wild type for this SNP (Figure 2, Table 4). Sperm produced by men with the GT genotype for rs12676 traveled greater distances at a faster rate and had more tortuous paths than sperm collected from men who were GG for this SNP. No differences between sperm from heterozygous and homozygous subjects were detected.

**Table 4:** Significance values for sperm motility analyses.

SNP	Motility measure	Overall p-value	Specific comparison		
rs12676			GG vs. GT	GG vs. TT	GT vs. TT
	MVUS	0.003	0.001 (GT>GG)	0.047 (TT>GG)	N.S.
	VCL	0.002	0.0006 (GT>GG)	0.02 (TT>GG)	N.S.
	VSL	N.S.	N.S.	N.S.	N.S.
	MT	<0.0001	0.0001 (GT>GG)	0.0009 (TT>GG)	N.S.

rs1025689			GG vs. GC	GG vs. CC	GC vs. CC
	MVUS	<0.0001	<0.0001 (GC>GG)	<0.0001 (CC>GG)	N.S.
	VCL	<0.0001	<0.0001 (GC>GG)	<0.0001 (CC>GG)	N.S
	VSL	<0.0001	<0.0001 (GC>GG)	0.005 (CC>GG)	N.S
	MT	0.02	N.S.	0.69	0.01 (CC>GC)

Statistical analyses were conducted as described in the Methods section. N.S, not significant.

**Figure 3:**
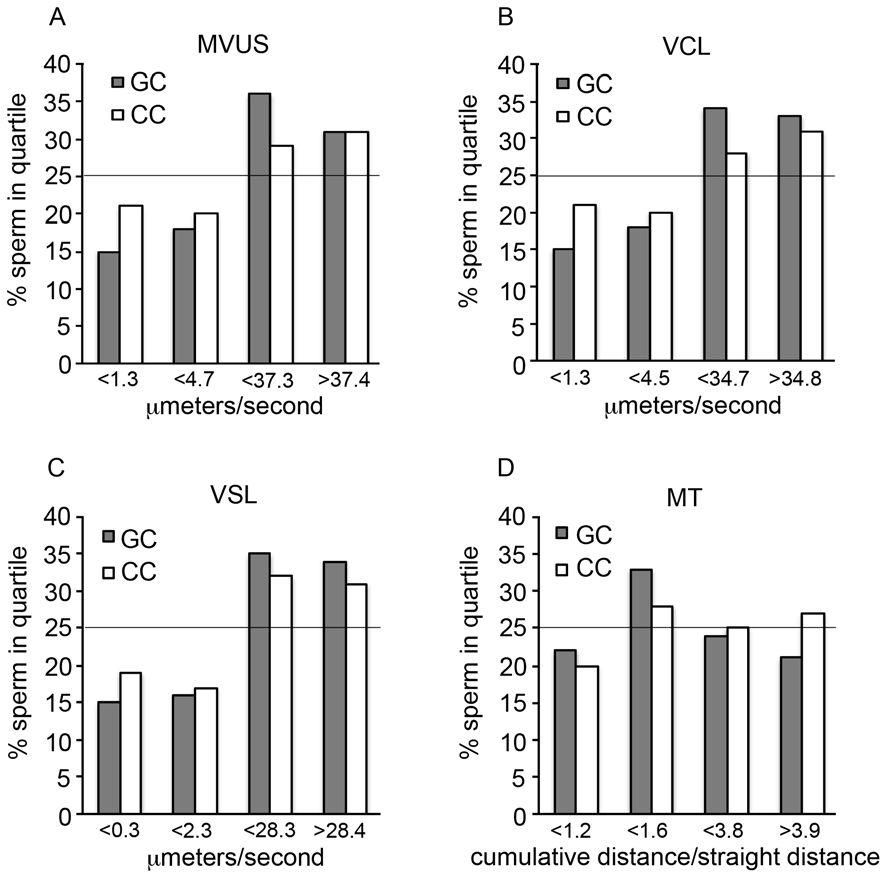
Motility characteristics change with rs1025689 genotype. Mean velocity (MVUS, *A*), curvilinear velocity (VCL, *B*), straight-line velocity (VSL, *C*) and mean tortuosity (MT, *D*) were determined for sperm. Sperm were prepared and motility characteristics measured as described in the Methods section. N = 1708 (GG), 2709 (GC), 1719 (CC) sperm. Statistical analyses were performed as described in the Methods section; refer to Table 4 for p-values. X-axis values indicate the upper limit of each quartile except for last label, which indicates the lower limit of the fourth quartile; the line at 25% represents values for sperm from GG subjects.

Sperm from men who were CC for rs1025689 had increased average velocity as well as curvilinear and straight line velocity when compared to sperm collected from men who were wild type for this SNP (Figure 3, Table 4). Men who were homozygous for rs1025689 produced sperm that traveled in more tortuous paths (i.e. less progressively motile) as compared to sperm from men who were heterozygous for this SNP. Subjects who were GC for rs1205689 produced sperm with higher measures of average velocity, curvilinear and straight line velocity compared to sperm from men with the GG genotype. Tortuosity was decreased in sperm from heterozygous men compared to sperm from men who were CC for rs1025689.

### Sperm mitochondrial ultrastructure and energy levels

**Figure 4:**
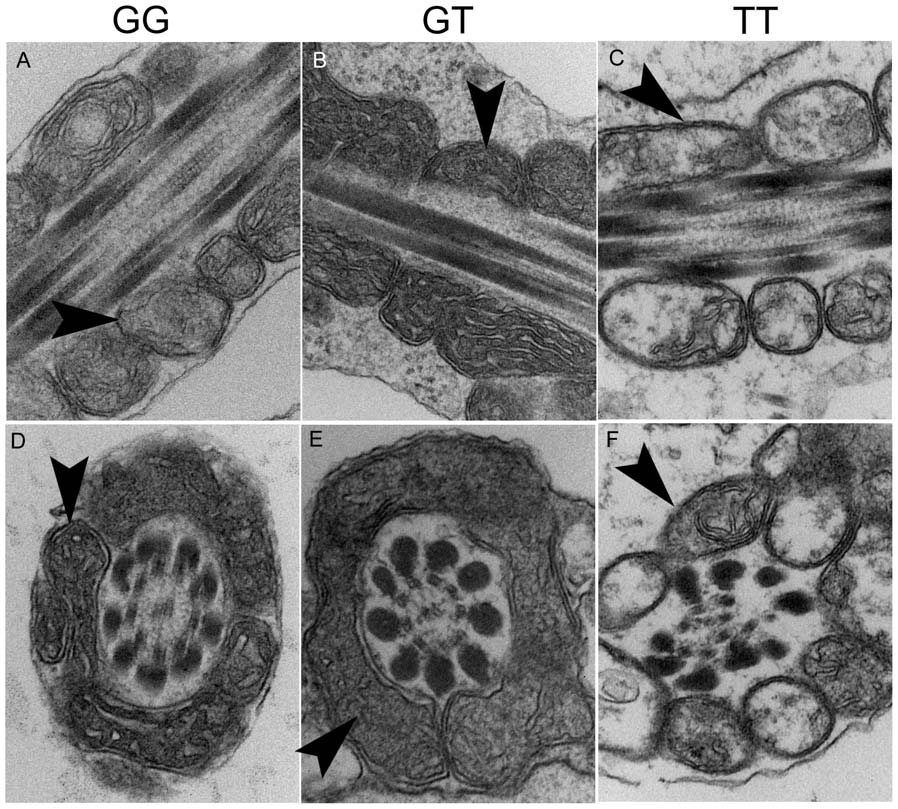
rs12676 TT genotype is associated with dysmorphic mitochondrial structure in sperm. Sperm were fixed and processed for transmission electron microscopy as described in the Methods section. Longitudinal and cross-sectional sections of sperm were examined for mitochondria structure anomalies. Representative images for rs12676 genotypes (GG, panel *A* and *D*; GT, panel *B* and *E*; TT, panel *C* and *F*) are shown. N = 5 per genotype. Micrographs shown are at 80,000× magnification and arrows indicate mitochondria.

**Figure 5:**
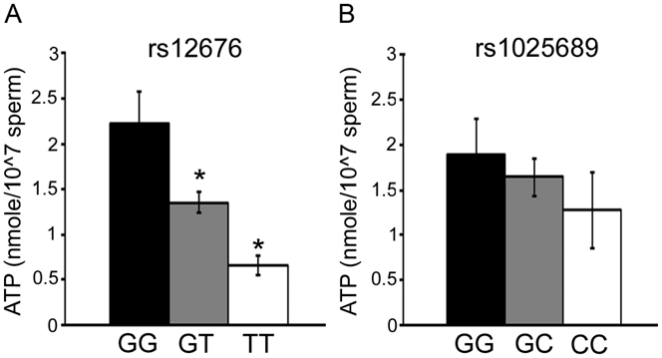
The rs12676 minor T allele is associated with decreased ATP concentrations in sperm. ATP concentrations were determined in sperm using a commercially available bioluminescent assay kit and ATP concentration was normalized to number of sperm assayed. *A*, Men who were heterozygous or homozygous for the rs12676 variant T allele have reduced ATP concentrations in their sperm. N = 17 (GG), 18 (GT) and 5 (TT). * indicates difference from GG by ANOVA and Tukey-Kramer HSD, p-value <0.05. *B*, ATP concentrations are not different with rs1025689 genotype. N = 10 (GG), 21 (GC) and 9 (CC). Data presented are mean ± SEM.

**Figure 6:**
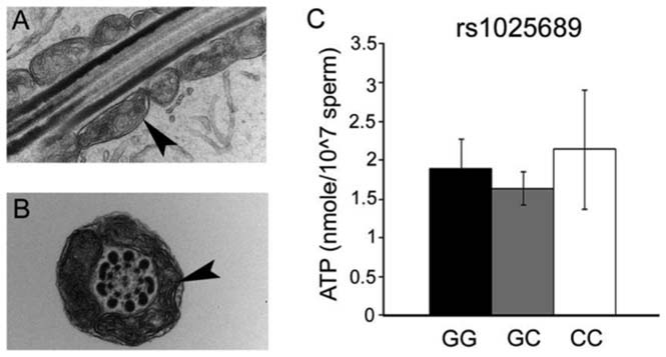
Sperm mitochondrial morphology and ATP concentrations are not changed in samples from men who are homozygous for rs1025689, but not rs12676. *A.* Representative longitudinal section of sperm midpiece. *B.* Representative cross-sectional section of sperm midpiece. Arrows indicate mitochondria. *C.* ATP concentrations as measured previously; “CC" group only contains data from men who are CC for rs1025689 and not TT for rs12676. Data presented are mean ± SEM. N = 9 (GG), 22 (GC) and 5 (CC).

Abnormal mitochondrial structure was observed in sperm collected from men who have two copies of the rs12676 minor allele (Figure 4). Mitochondria in the midpiece of these sperm appeared swollen with disordered cristae structure. Sperm from men with one allele of rs12676 (GT) have 40% less ATP than sperm produced by men who are GG for this SNP (Figure 5). Men carrying two alleles of the SNP (TT) produce sperm with 73% less ATP than men who are GG. 83% of subjects who were homozygous for rs12676 were also homozygous for rs1025689, but when mitochondrial morphology and ATP concentrations were analyzed in men who were homozygous for rs1025689 only, and we found there was no relationship between rs1025689 genotype and these measures (Figure 6).

### CHDH protein expression

**Figure 7:**
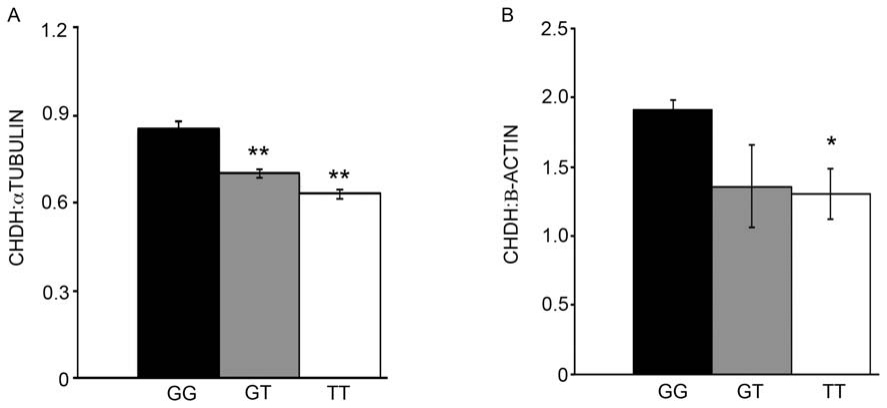
Expression of CHDH protein is decreased in sperm and primary hepatocytes from humans who are heterozygous or homozygous for the rs12676 SNP. The abundance of CHDH protein in sperm (A) and primary hepatocyte (B) lysates was measured by western blot. Blots were probed for ∝-TUBULIN (sperm) or β-ACTIN (hepatocytes) and data presented are the mean ± SEM of the ratio of CHDH: ∝-TUBULIN or CHDH:β-ACTIN protein. Statistical differences were tested by ANOVA and Student's *t* test. * indicates different from GG, p-value <0.05. ** indicates different from GG, p-value <0.01. N = 3 per genotype.

CHDH protein expression, relative to ∝-TUBULIN expression, was decreased in sperm produced by men who had one or two alleles of rs12676 (Figure 7A). This effect was observed in non-reproductive tissues as well; individuals who harbor the TT rs12676 genotype had a lower CHDH protein in their hepatocytes compared to individuals who were GG for this SNP (Figure 7B). The CHDH protein levels in GT hepatocytes were not significantly changed from either GG or TT hepatocytes. Both ∝-TUBULIN and β-ACTIN protein expression was not changed among the genotypic groups (not shown).

### Plasma choline metabolite concentrations

Choline metabolite concentrations in plasma and sperm were measured using LC-ESI-IDMS. Men who were homozygous for rs12676 minor allele had a small, but significant increase in plasma free choline concentrations (Table 5). There were no differences in plasma betaine, PtdCho or SM among the other rs12676 or rs1025689 genotypes. There was no effect of rs12676 genotype on sperm choline metabolite concentrations. Sperm from men who were CC for rs1025689 produced sperm with decreased betaine, GPCho and PtdCho concentrations compared to sperm produced by men who were GC for this SNP.

**Table 5:** Choline metabolite concentrations in plasma and sperm.

Plasma				
rs12676		GG	GT	TT
	Betaine	62.2±2.5	65.1±2.8	75.3±6.4
	Choline	11.0±0.4	10.5±0.5	13.0±1.0*
	PtdCho	1802.4±60.1	1938.9±70.3	1964.6±157.2
	SM	727.8±26.0	780.0±30.0	759.6±67.2

rs1025689		GG	GC	CC
	Betaine	57.4±4.0	66.0±2.6	66.3±3.2
	Choline	10.4±0.7	18.9±0.4	10.8±0.6
	PtdCho	1872.3±99.8	1924.6±63.5	1781.5±78.9
	SM	728.4±42.8	766.4±27.2	741.1±33.8

Sperm				
rs12676		GG	GT	TT
	Betaine	0.9±0.3	1.21±0.3	0.35±0.4
	Choline	0.24±0.0	0.2±0.0	0.1±0.1
	GPCho	1.3±0.3	1.7±0.2	1.0±0.7
	PtdCho	3.1±0.2	3.4±0.2	3.0±0.5

rs1025689		GG	GC	CC
	Betaine	0.8±0.3	1.5±0.3	0.4±0.3†
	Choline	0.2±0.1	0.2±0.0	0.1±0.0
	GPCho	1.4±0.4	2.0±0.2	0.7±0.3†
	PtdCho	3.1±0.3	3.7±0.2	2.7±0.2†

Concentrations of choline and its metabolites were measured in plasma collected during the Day 1 clinic visit as described in Methods. For rs12676 *N* = 40 (GG), 30 (GT) and 6 (TT). For rs1025689 *N* = 15 (GG), 37 (GC) and 24 (CC). Data are expressed nmol/L and are presented as mean ± SEM. ANOVA and Tukey-Kramer HSD were used to detect statistical differences among groups.

* indicate p<0.05. Choline metabolite concentrations were measured in 3 million sperm from each subject who provided a semen sample on Day 2. For rs12676 *N* = 17 (GG), 18 (GT) and 4 (TT). For rs1025689 *N* = 11 (GG), 17 (GC) and 11 (CC). Data are expressed as nmol/3million sperm and are presented as mean ± SEM. ANOVA and Tukey-Kramer HSD were used to detect statistical differences among groups.

† indicates different from rs1025689 GC genotype, p<0.05.

## Discussion

We previously reported the novel finding that *Chdh* deletion in mice resulted in male infertility due to compromised sperm motility. We now present evidence demonstrating that this discovery translates, in part, to humans. A common SNP in the human *CHDH* gene, rs12676, is associated with altered sperm motility patterns, dysmorphic mitochondrial ultrastructure and a significant reduction in ATP concentrations in sperm. Humans who were heterozygous or homozygous for this SNP had less CHDH protein in their sperm. Interestingly, this association also was observed in hepatocytes from individuals who were homozygous for this SNP. Further studies are required to determine whether this is a result of decreased *CHDH* mRNA translation or increased CHDH protein degradation. Either way, this data suggests that rs12676 is a functional SNP, or that it is a tag SNP that marks a functional haplotype of the *CHDH* gene. In addition, rs1025689, a SNP located in the adjacent *IL17βR* gene that is highly associated with increased susceptibility to dietary choline deficiency in men, is correlated with changes in sperm motility patterns and reduction of betaine, GPCho and PtdCho concentrations in sperm.

The allele frequency distributions of the screened population for both rs12676 and rs1025689 were in agreement with previously reported frequencies [35], [36], [40]. The average age among the genotypic groups was not significantly different; therefore, we conclude that any changes detected in semen characteristics or sperm cell function were not due to differences in age among the groups. The rs12676 TT genotype group reported the lowest number of biological children per subject (0.33) and the highest rate of semen abnormality/infertility diagnosis (33%) of all groups, which suggests that men with the TT genotype may be subfertile.

Interestingly, subjects who were homozygous for rs12676 had higher free choline concentrations in their plasma. Although plasma choline is not a direct measure of choline concentrations within tissues, it is a reflection of tissue concentrations. An increase in free choline would be expected with a decrease in CHDH activity as less choline would be converted to betaine. We did not detect a decrease in betaine in these men, probably because we did not require that the subjects to fast prior to their blood draw and betaine can be obtained from the diet.

Sperm from men who harbor at least one minor allele of rs12676 were less progressively motile, as indicated by an increase in curvilinear velocity and tortuosity (Figure 2, Table 4). Mean velocity was significantly increased in sperm from heterozygous and homozygous subjects. Hyperactivated sperm are those displaying motility patterns characterized by vigorous, non-linear trajectories [42] and are in contrast to progressively motile sperm which move forward in a somewhat linear path [43]. Sperm released from the cauda epididymis typically are progressively motile at first and transition to hyperactivated motility when incubated in media formulated to support this process [42]. Sperm collected from the female reproductive tract are generally hyperactivated [42]. Although the HTF medium used to wash the sperm in our experiments is designed to mimic the environment of the female reproductive tract, we cannot definitively state whether the observed changes in motility patterns with respect to rs12676 genotype were the result of premature hyperactivation or a decrease in progressive motility. HTF media provides all metabolic substrates required for capacitation and hyperactivation and it is reasonable to assume that the sperm used for the motility analyses could have achieved hyperactivation. Sperm produced by men who were GC for rs1025689 were more progressively motile than sperm from men who were either GG or CC for this SNP.

rs12676, but not rs1025689, was associated with dysmorphic mitochondrial structure (Figure 4 and Figure 6). ATP concentration was inversely correlated, in a dose-dependent manner, with the number of rs12676 minor alleles (Figure 5). Although the exact mechanism causing these changes remains unknown, these results are very similar to those we observed in the *Chdh^−/−^* mice [21]. ATP is required for sperm to be motile, but considerable controversy surrounds the source of the ATP used for this function. Because mitochondria are only found in the sperm midpiece, there is some question as to whether ATP generated by oxidative phosphorylation (OXPHOS) can diffuse the length of the tail to supply substrate for the dynein ATPases [44]. A creatine phosphate shuttle system has been proposed that may traffic ATP from mitochondria through the tail, but experimental evidence supporting the existence of this mechanism is still lacking [45]. Alternatively, glycolytic enzymes are localized to the principal piece of the sperm tail, thus providing a source of ATP at the site in which it will be used [46], [47], [48], [49], [50]. In most species there is evidence that both pathways are active in sperm cells; however, the relative importance of each pathway differs among species. For example, OXPHOS-derived ATP supports bull and ram sperm motility [51], while mouse sperm has a definitive need for glycolytic generation of ATP for motility [46], [47], [48], [49], [50]. Surprisingly, sperm produced by *Chdh^−/−^* mice are characterized by a reduction in both mitochondrial oxygen consumption rates – an indication of OXPHOS activity – and glycolytic rates indicating that loss of CHDH function perturbs the energy homeostasis in these cells (Figure S2), thus resulting in an overall decrease in ATP concentration.

It is possible that the betaine molecule itself plays an important role in maintaining testicular and sperm cell function and, in particular, spermatic ATP concentrations. Dietary betaine supplementation of *Chdh^−/−^* male mice resulted in total restoration of ATP levels in sperm and an increase in progressive motility of these cells [21]. Betaine is an organic osmolyte used by cells for protection during times of osmotic stress [52], [53]. Sperm mature as they move from the lumen of the testis and through the caput, corpus and cauda regions of the epididymis [54]. During transit, sperm accumulate molecules found within the epididymal environment including organic osmolytes such as glycerophosphocholine and carnitine [54]. We measured betaine concentrations in the mouse epididymis and found levels 10 times greater than those measured in liver (Table S1). The epididymal environment is relatively hyperosmotic (∼340 mmol/kg, [55]). In comparison, the osmolality of unliquified whole semen and of the fluid in the female reproductive tract is approximately 276–302 mmol/kg [56], [57] indicating that epididymal sperm experience an osmotic “challenge" within the male urethra [55]. An inability to regulate volume in response to the varied osmotic environments would render sperm susceptible to swelling which can impair motility [57].

Evidence exists that links betaine to sperm motility. Preserving normal motility characteristics in sperm that have been frozen and thawed is an active area of research both in the fields of human andrology and veterinary animal husbandry. Kroskinen, et al [58] and Sanchez-Partida et al [59] reported increased sperm motility in thawed sperm when betaine was added to the cryopreservation media. It is hypothesized that betaine may directly interact with membrane lipids and proteins, altering the hydration status of these molecules, and thus protecting them through the freeze/thaw cycle [60].

Changes in sperm membrane phospholipid composition may also contribute to abnormal sperm cell function. For example, *Chdh^−/−^* sperm have half as much PtdCho and GPCho (a metabolite formed from PtdCho) when compared to *Chdh^+/+^* sperm (Figure S3). Increased osmotic stress due to a lack of betaine may account for this via activation of phospholipase A_2_ (PLA_2_). PLA_2_, highly expressed in sperm [61] and activated by osmotic stress [62], catalyzes the hydrolysis of the fatty acid in the sn-2 position of PtdCho resulting in the release of the fatty acid and generation of lyso-PtdCho [63]. The fatty acids in this position tend to be docosahexanoic acid (DHA) and arachidonic acid (AA). All of these molecules have been shown to inhibit sperm motility [63]. Sperm produced by men who were CC for rs1025689 contained less betaine, PtdCho and GPCho (Table 5).

rs1025689 is a synonomous SNP located in the coding region of human *IL17βR*. In a separate study, men who were homozygous for the minor C allele, were more likely to develop signs of liver or muscle dysfunction when ingesting a choline-deficient diet. Because the presence of this SNP does not result in an amino acid change, it most likely tags a functional haplotype within this gene. *CHDH* and *IL17βR* are situated in a head-to-head orientation on opposite strands on human chromosome 3 and mouse chromosome 14 [64], [65]. According to available data, rs12676 and rs1025689 are not in linkage disequilibrium; however, we noted a high degree of concurrence of these SNPs within our study population. Although we are not aware of any reports of copy number variations in the choline dehydrogenase gene locus, it is also possible this occurs and should be investigated in future studies. Because they share a promoter region, it is likely that transcriptional regulation of *CHDH* and *IL17βR* are similar. For example, transcription of these genes is enhanced by estrogen; an estrogen response element is located within the shared promoter region [64]. Aberrant expression of *CHDH* and *IL17βR* has been associated with breast cancer survival prognosis [64], [65]. Ours is the first report linking the function of this chromosomal region to male sperm cell function.

In this study, rs12676 is the primary predictor of abnormal sperm mitochondrial morphology and ATP concentration and this is strengthened by the finding that CHDH protein expression is decreased in sperm from men who were GT or TT, and hepatocytes from individuals who were TT, for this variation. No changes in mitochondrial ultrastructure or ATP level were detected in sperm from individuals who were CC for rs1205689, but not TT for rs12676 (Figure 6). Together, this evidence indicates that altered CHDH activity due to rs12676 genotype may be an underlying cause of iodiopathic male factor infertility in men. This is an especially interesting finding because deficits in CHDH function may be overcome by dietary supplementation with betaine. Indeed, as noted above, sperm motility and ATP concentration were improved in *Chdh^−/−^* male mice ingesting betaine-supplemented drinking water [21].

## Supporting Information

Figure S1 **Health questionnaire form.** All subjects completed the health questionnaire form during the screen visit.
(PDF)

Figure S2 **Oxygen consumption rates (OCR) and extracellular acidification rates (ECAR) in** ***Chdh^+/+^*** **and** ***Chdh^−/−^*** **sperm.** Sperm were released from the cauda epididymides from *Chdh^+/+^* and *Chdh^−/−^* male mice into modified HFT media. Modified HTF did not contain sodium bicarbonate, but did contain 1 mM Sp-5,6-dichloro-1- beta-D- ribofuranosylbenzimidazole-3′,5′-monophosphorothioate (Sp-5,6-DCl-cBiMPS), a cell permeable cAMP analog, and 1 mM 3-isobutyl-1-methylxanthine (IBMX), a phosphodiesterase inhibitor. Together, these additives are a substitute for sodium bicarbonate in the HTF media. Sodium bicarbonate signaling increases cAMP levels in sperm which is a signal necessary for achieving capacitation. 4 million sperm were aliquoted into each well of a 24 well Seahorse Bioscience tissue culture plate. Modified HTF media was added so that the final volume in each well was 500 µL. The Seahorse analyzer was calibrated and equilibrated according to manufacturer's instructions. OCR and ECAR measurements were recorded over the course of ∼100 minutes following a protocol of mixing for 2 minutes, waiting for 3 minutes and measuring for 4 minutes. N = 5 (*Chdh^+/+^*) and 3 (*Chdh^−/−^*); data are mean ± SEM for each genotype group.
(DOCX)

Figure S3 **PtdCho and GPCho are decreased in** ***Chdh−/−*** **sperm.** *Chdh^+/+^* and *Chdh^−/−^* male mice, at least 10 weeks of age, were anesthetized using Isofluorane until they no longer responded to a pain stimulus. Sperm were released into HTF media from the cauda epididymis as described previously. After 2 hour incubation in HTF, 4 million sperm from each animal were pelleted in a 1.5 mL microcentrifuge tube and processed for choline metabolite analysis as described previously. N = 5 animals per genotype. Data are presented as mean ± SEM. Student's *t* test was used to test for statistical differences between genotypic groups. ** indicate p-value>0.01. Only metabolites in which there were significant changes are shown.
(DOCX)

Table S1 **Choline metabolite concentrations in** ***Chdh*** ***^+/+^*** **and** ***Chdh*** ***^−/−^*** **epididymis.** *Chdh^+/+^* and *Chdh^−/−^* male mice, at least 10 weeks of age were anesthetized using Isofluorane until they no longer respond to a pain stimulus. Caput, corpus and cauda epididymides were collected; both whole epididymides were pooled together for each animal. Tissues were snap frozen in liquid nitrogen, sonicated for 1 minute and processed for choline metabolite measurements as described [226]. N = 6 (*Chdh^+/+^*) and 3 (*Chdh^−/−^*). Data are presented as mean ± SEM. Student's t test was used to test for statistical differences between genotypic groups. * indicate p-value >0.05, ** indicate p-value >0.01.
(DOCX)
