# Supplementary material for: Choline Dehydrogenase Polymorphism rs12676 Is a Functional Variation and Is Associated with Changes in Human Sperm Cell Function
Source: PLoS One. 2012 Apr 27;7(4):e36047. doi: 10.1371/journal.pone.0036047 (PMC3338626; doi:10.1371/journal.pone.0036047)
Supplement: Figure S1 — Health questionnaire form. All subjects completed the health questionnaire form during the screen visit. (PDF) [file pone.0036047.s001.pdf]

Subject ID # _______

Choline dehydrogenase and sperm motility in men

Medical History Questionnaire

Name ____ Date ________________________

Date of birth _________________ Marital Status __________________ Number of children_________

Were any of your children conceived using assisted reproductive technology due to male factor infertility? Yes / No

Have you ever been diagnosed with a fertility problem? Yes / No

If "yes" please circle all that apply:

Low sperm count Low sperm motility Increased semen viscosity

Decreased semen viscosity Abnormal sperm shape Increased semen volume

Decreased semen volume Abnormal semen pH Abnormal liquefaction time

Abnormal number of white blood cells in semen

Other, please explain ____

____

Were you ever treated for a fertility problem? Yes / No

If "yes", please describe treatment: ____

____

Was your condition improved with treatment? Yes / No

Do you have male family members with fertility problems? Yes / No

If "yes", what is their relationship to you (brother, father, etc)? ____________________

Are you currently taking any medication? If "yes", please specify. _________________________

____

Are you taking any medication known to alter fertility or sperm function? Yes / No

Please specify. ____

Are you currently being treated for a medical condition? Yes / No

If "yes", please specify. ____

____

Do you have a chronic medical condition? Yes / No

If "yes", please specify____
