# Supplementary material for: Choline Dehydrogenase Polymorphism rs12676 Is a Functional Variation and Is Associated with Changes in Human Sperm Cell Function
Source: PLoS One. 2012 Apr 27;7(4):e36047. doi: 10.1371/journal.pone.0036047 (PMC3338626; doi:10.1371/journal.pone.0036047)

**Figure S2: Oxygen consumption rates (OCR) and extracellular acidification rates (ECAR) in *Chdh+/+* and *Chdh-/-* sperm**


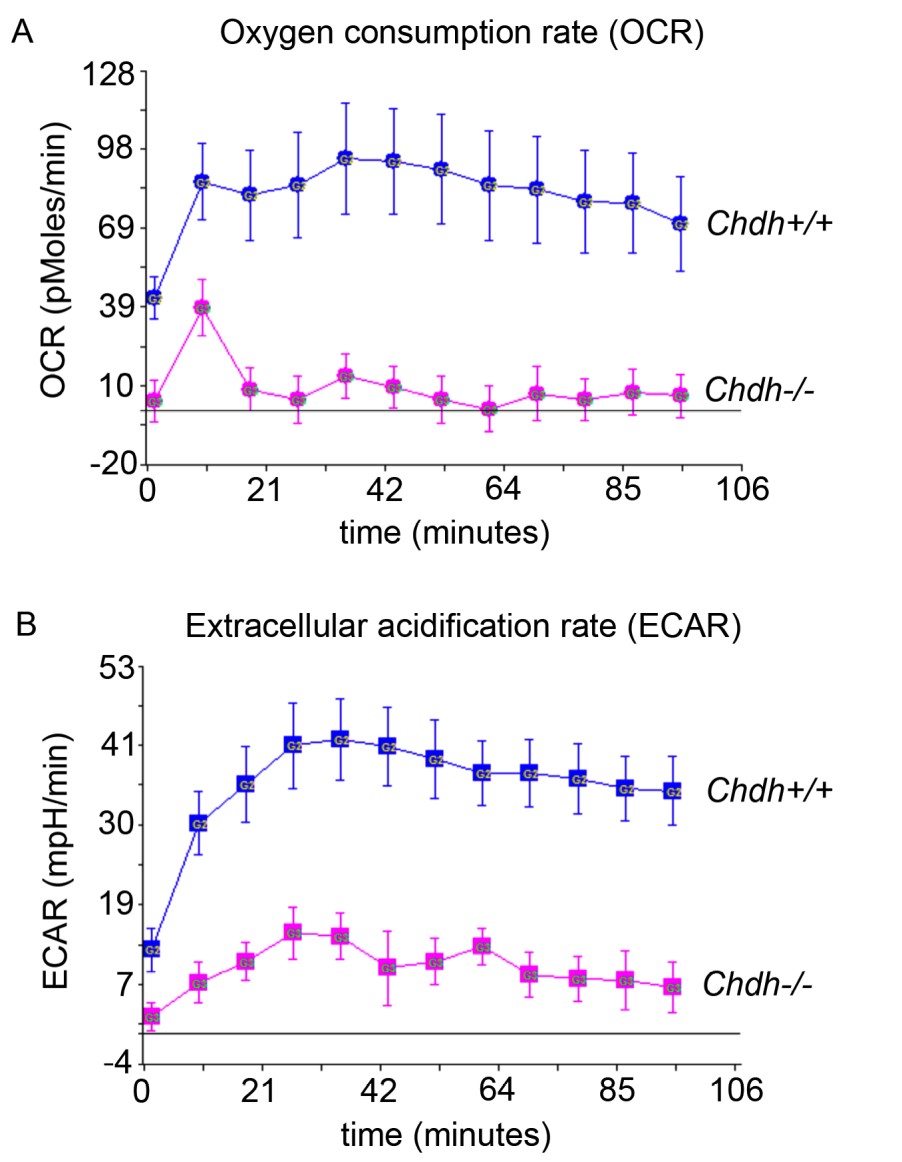

Supplement: Figure S2 — Oxygen consumption rates (OCR) and extracellular acidification rates (ECAR) in Chdh+/+ and Chdh−/− sperm. Sperm were released from the cauda epididymides from Chdh+/+ and Chdh−/− male mice into modified HFT media. Modified HTF did not contain sodium bicarbonate, but did contain 1 mM Sp-5,6-dichloro-1- beta-D- ribofuranosylbenzimidazole-3′,5′-monophosphorothioate (Sp-5,6-DCl-cBiMPS), a cell permeable cAMP analog, and 1 mM 3-isobutyl-1-methylxanthine (IBMX), a phosphodiesterase inhibitor. Together, these additives are a substitute for sodium bicarbonate in the HTF media. Sodium bicarbonate signaling increases cAMP levels in sperm which is a signal necessary for achieving capacitation. 4 million sperm were aliquoted into each well of a 24 well Seahorse Bioscience tissue culture plate. Modified HTF media was added so that the final volume in each well was 500 µL. The Seahorse analyzer was calibrated and equilibrated according to manufacturer's instructions. OCR and ECAR measurements were recorded over the course of ∼100 minutes following a protocol of mixing for 2 minutes, waiting for 3 minutes and measuring for 4 minutes. N = 5 (Chdh+/+) and 3 (Chdh−/−); data are mean ± SEM for each genotype group. (DOCX) [file pone.0036047.s002.docx]
