# Supplementary figures and images for: Choline Dehydrogenase Polymorphism rs12676 Is a Functional Variation and Is Associated with Changes in Human Sperm Cell Function
Source: PLoS One. 2012 Apr 27;7(4):e36047. doi: 10.1371/journal.pone.0036047 (PMC3338626; doi:10.1371/journal.pone.0036047)

**Figure S3: PtdCho and GPCho are decreased in *Chdh-/-* sperm**

**A**


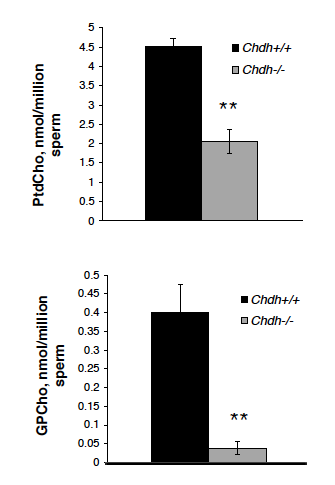


**B**

Supplement: Figure S3 — PtdCho and GPCho are decreased in Chdh−/− sperm. Chdh+/+ and Chdh−/− male mice, at least 10 weeks of age, were anesthetized using Isofluorane until they no longer responded to a pain stimulus. Sperm were released into HTF media from the cauda epididymis as described previously. After 2 hour incubation in HTF, 4 million sperm from each animal were pelleted in a 1.5 mL microcentrifuge tube and processed for choline metabolite analysis as described previously. N = 5 animals per genotype. Data are presented as mean ± SEM. Student's t test was used to test for statistical differences between genotypic groups. ** indicate p-value>0.01. Only metabolites in which there were significant changes are shown. (DOCX) [file pone.0036047.s003.docx]
