# Supplementary material for: Choline Dehydrogenase Polymorphism rs12676 Is a Functional Variation and Is Associated with Changes in Human Sperm Cell Function
Source: PLoS One. 2012 Apr 27;7(4):e36047. doi: 10.1371/journal.pone.0036047 (PMC3338626; doi:10.1371/journal.pone.0036047)
Supplement: Table S1 — Choline metabolite concentrations in Chdh +/+ and Chdh −/− epididymis. Chdh+/+ and Chdh−/− male mice, at least 10 weeks of age were anesthetized using Isofluorane until they no longer respond to a pain stimulus. Caput, corpus and cauda epididymides were collected; both whole epididymides were pooled together for each animal. Tissues were snap frozen in liquid nitrogen, sonicated for 1 minute and processed for choline metabolite measurements as described [226]. N = 6 (Chdh+/+) and 3 (Chdh−/−). Data are presented as mean ± SEM. Student's t test was used to test for statistical differences between genotypic groups. * indicate p-value >0.05, ** indicate p-value >0.01. (DOCX) [file pone.0036047.s004.docx]

**Supplemental Table 1: Choline metabolite concentrations in *Chdh+/+* and *Chdh-/-* epididymis**

|  |  | **Betaine** | **Choline** | **GPCho** | **PCho** | **PtdCho** | **SM** |
| --- | --- | --- | --- | --- | --- | --- | --- |
|  |  | (nmol/g) | (nmol/g) | (nmol/g) | (nmol/g) | (nmol/g) | (nmol/g) |
| **Epididymis** |  |  |  |  |  |  |  |
|  | ***Chdh^+/+^*** | 2602 ± 688 | 1442 ± 459 | 20582 ± 1389 | 952 ± 60 | 14085 ± 917 | 2556 ± 104 |
|  | ***Chdh^-/-^*** | 2 ± 2* | 2235 ± 497* | 32405 ± 6208 | 798 ± 172 | 12233 ± 2203 | 2525 ± 168 |
